# Tracking of Physical Fitness of Primary School Children in Trier: A 4-Year Longitudinal Study

**DOI:** 10.1155/2018/7231818

**Published:** 2018-04-22

**Authors:** Andreas Roth, Steffen C. E. Schmidt, Ilka Seidel, Alexander Woll, Klaus Bös

**Affiliations:** ^1^Research Centre for School Sports and the Physical Education of Children and Young Adults, Karlsruhe Institute of Technology, Karlsruhe, Germany; ^2^Institute of Sport and Sport Science, Karlsruhe Institute of Technology, Karlsruhe, Germany

## Abstract

**Objective:**

The aim of this study is to measure the motor development and tracking of physical fitness (PF) components of primary school children of Trier in Germany.

**Methods:**

Two longitudinal cohorts, of 1768 children (915 f, 853 m) aged 5–11, were measured. In longitudinal cohort 1, a total of 116 female and 137 male participants aged 6.80 ± 0.42 years at baseline were measured four times from grade 1 to grade 4 (response: 40.4%). Participants of longitudinal cohort 2 (166 f, 149 m; 6.70 ± 0.36 years at baseline, response: 42.6%) were examined three times from grade 1 to grade 3 with the German Motor Test 6–18 (DMT 6–18).

**Results:**

Physical fitness increased significantly over time in all test tasks except flexibility. Gender-specific differences were found in 20 m sprint, 6-minute run, balancing backwards, jumping sideways, and stand and reach. 74.4% of PF stability coefficients were moderate (*r* = 0.30 to 0.60). Stability of PF declined with increased time frames. Tracking was lower in girls than in boys. Flexibility showed the highest stability among PF variables (*r* > 0.50). BMI showed the overall highest stability coefficient with *r* > 0.7.

**Conclusions:**

Gender-specific differences of PF were obvious but cannot always be secured statistically in primary school. Tracking was only moderate. Variability in the timing and speed of the adolescent growth spurt and sexual maturation influence stability of PF. Results from longitudinal cohort 2 largely confirm those from longitudinal cohort 1.

## 1. Introduction

Regular physical activity (PA) and physical fitness (PF) are important factors for the health and social development of children and adolescents. PF is considered one of the most important health markers [1]. It is defined as a set of personal properties (i.e., cardiorespiratory endurance, skeletal muscle endurance, skeletal muscle power, flexibility, agility, balance, reaction time, and body composition) which people inherit or advance to perform PA. Cardiorespiratory endurance, muscular strength, endurance, body composition, and flexibility are referred to as health-related fitness. Balance, coordination, speed, agility, and power are often characterized as performance-related fitness [2].

There is consensus that high PF positively influences the physical and psychological health of children and adolescents. Studies show that cardiorespiratory fitness as well as muscular fitness reduce overall and abdominal obesity and decrease cardiovascular risk factors [1, 3–6]. Moreover, cardiorespiratory fitness and muscular fitness can have a positive influence on mental health [1, 3] and are also positively associated with academic achievement [7]. An increase in PF is positively associated with bone health and higher quality of life after cancers and chemotherapy-induced treatments [1].

Motor performance, measured by PF components in childhood, is a process of change which is determined by development-related changes and by training-dependent performance improvements. In the early school years pupils are subject to extensive physical changes. Internal organs develop their full functionality, the body undergoes widespread sequential changes, and the central nervous system matures [8].

During physical development of childhood speed, endurance and strength increase in both genders [8–10]. However, especially at primary school age, the time of motor ability development varies due to different maturation levels [11]. The development of speed and endurance is quicker than the development of muscular strength. Coordination is based on a large inter- and intraindividual range of performance because of the interplay of complex external and internal factors [8]. At primary school age, girls have better flexibility than boys [8, 9].

In adolescence, motor performance continues to improve and sex differences become more considerable. In speed, endurance, and strength, boys often achieve higher results than girls. These differences become significant at the age of 12 to 13 years [11]. Due to various developments of endocrinological processes in the context of puberty, the effects of training in males increase enormously, especially in strength [11, 12]. PF usually reaches its peak in late adolescence or early adulthood. Girls reach the plateau of conditionally based testing tasks earlier than boys [13].

In general, numerous internal and environmental factors can lead to several instabilities in individual development. Reported childhood development is heavily dependent on the sample and the test battery used. Particularly with coordination tasks, there are often low correlations between the results of different tasks [8].

Due to individual differences in development, future motor performance is hard to predict. Measuring a sample at different points in time is called tracking and provides a coefficient for the longitudinal stability of a certain variable [14]. These coefficients of motor performance stability demonstrate if fit children are also fit at a later point in life. This is an important question in talent promotion, but it also allows the early detection of clumsy and motor deficit children.

It has been confirmed that tracking coefficients depend on the time frame between different measures. In general, the correlation increases with a shorter time frame. The height of the coefficient also depends on the age of the sample at baseline [14]. At young school age the coefficients are mostly lower than after puberty [15]. Coefficients also depend on sample size, the claimed motor ability, and motor testing tasks used [15]. Studies show that boys reach slightly higher coefficients than girls [10]. There are also differences in the stability of anthropometric and motor skills, and Body Mass Index is usually more stable than PF [16].

PF components were tracked from childhood to adolescence [16–19], during adolescence [20], or from childhood or adolescence to adulthood [21–24], and during childhood [10, 25, 26].

Falk et al. [10] examined the tracking of field-assessed fitness components for 319 pupils (116 f 203 m) from the second grade, which corresponds to the age of 6-7 years, to the sixth grade. The correlations of fitness components over the 4-year period in both sexes varied between *r* = .36 and .66. Stability was generally lower in girls than in boys.

The study of McMillan and Erdmann [25] tracked health-related fitness components for 409 boys and 409 girls from kindergarten (6.1 ± 0.3 years) to the fifth grade. The correlations from kindergarten to the second grade lie in the range of *r* = .39 to *r* = .82, and from kindergarten to the third grade from *r* = .37 to *r* = .84. Vandorpe and colleagues [26] examined 371 children between six and nine years of age at baseline regarding motor coordination and sports club participation in three consecutive years. Correlation coefficients ranging from .66 (6–8 years) to .87 (7–9 years) revealed that coordination of children is a highly stable factor in this study.

The main objective of the TrieKis project was to carry out a screening of all primary school children of Trier, in order to identify the students' strengths and weaknesses at different measurements points.

In the following paper the motor development and stability of motor performance in primary school age are the focus.

The main research questions are as follows:

- What is the course of motor performance in primary school?
- How stable is motor performance in primary school?

## 2. Research Methods

### 2.1. Study Sample and Design

The data was collected during a community-based, longitudinal study in Trier (population approximately 115,000) with four measurements, during the school years 2008/2009, 2009/2010, 2010/2011, and 2011/2012. Participants were recruited from 23 out of 24 in Trier available primary schools in Trier and were tested mostly in physical education lessons. The study was a joint project of the sports association in Rhineland-Pfalz, the Supervision and Services Directorate in Trier, the Physical Education College of Trier, and the Research Center for School Sports and Physical Education of Children and Young Adults (FoSS).

Participation was mandatory for the pupils, and parents were informed early and could refuse participation for their children. The tests were organized and carried out by trained instructors of the Physical Education College of Trier in cooperation with FoSS.

A total of 1768 different subjects (915 f, 853 m) aged 5–11 were tested 4266 times over the course of the study. The total numbers of participants for each of the four measurement points were 2008/2009: 623, 2009/2010: 1404, 2010/2011: 1335, and 2011/2012: 1335. In the early stages of the study 623 pupils (313 f, 310 m) were measured in grade 1. That led to a total number of 252 (116 f. 137 m.; 6.80 ± 0.42 years at baseline) pupils who were tested at each of the four measurement points (longitudinal cohort 2008; LC 1). This represents 40.4% (37.1% girls; 44.2% boys) of the initial sample. In 2009/2010, 739 (381 f, 358 m) new pupils participated. From those, 315 (166 f, 149 m; 6.70 ± 0.36 years at baseline) were tested three times (longitudinal cohort 2009; LC 2). This represents 42.6% (43.6% girls; 41.6% boys) of the sample which started in 2009/2010. Descriptive statistics of the sample are shown in Table 1.

The sample shows representative characteristics regarding age, sex, anthropometrics, and PF of primary school children for a moderate-sized city in Germany. Since the sample was recruited from 23 out of 24 available primary schools in Trier, we assume that the sample is also representative in terms of social status and migration background.

### 2.2. Measures

#### 2.2.1. Physical Fitness

The German Motor Test (DMT 6–18) [13] was used to assess PF. One-week reliability of test tasks performed by a comparable team of trained instructors is on average *r* = .82. Test battery was successfully checked for validity [13].

Cardiorespiratory fitness was measured by the 6-minute run. Strength endurance of upper extremities was evaluated by number of push-ups in 40 seconds. Strength endurance of the torso muscles was evaluated by number of sit-ups in 40 seconds. Speed strength of lower extremities was checked by standing long jump. Action speed was evaluated by the 20 m sprint using a stop watch. Cross-motor coordination under time constraint was measured by jumping sideways. Backward balancing allowed the assessment of gross motor coordination during dynamic precision tasks. The number of steps on each beam was added. The stand and reach test was used for the assessment of trunk flexibility and the flexibility of the sciatic-crural muscle group [13].

In a 45-minute time frame, ten to twelve pupils were tested by a group of eight test instructors from the Physical Education College of Trier.

#### 2.2.2. Body Mass Index

Height was measured, without shoes, to the nearest 0.1 cm using a tape measure. Weight was measured standardized to the nearest 0.1 kg using a Korona Alva digital metric scale (Sundern, North Rhine Westfalia, Germany). Body Mass Index (BMI) has been accepted as a viable diagnostic tool for estimating fat mass in children and adolescents and was also measured in the study [27]. BMI can be calculated as body weight (kg)/(body size (m))^2^.

### 2.3. Statistical Analysis

Statistical analysis was performed using SPSS Statistics 24.0. Significance level was set to *p* < .05. To quantify stability, Pearson's correlation coefficients were calculated between grades. Tracking analyses included only subjects that participated in every measurement point. Significant PF changes over time and time*∗*sex interactions were analysed via repeated measurement ANOVA (rmANOVA) and *F*-value and partial eta^2^ effect sizes are reported.

## 3. Results

### 3.1. Descriptive Statistics

Descriptive statistics of LC 1 are shown in Table 2, and descriptive statistics of LC 2 are shown in Table 3.

**Figure 1:**
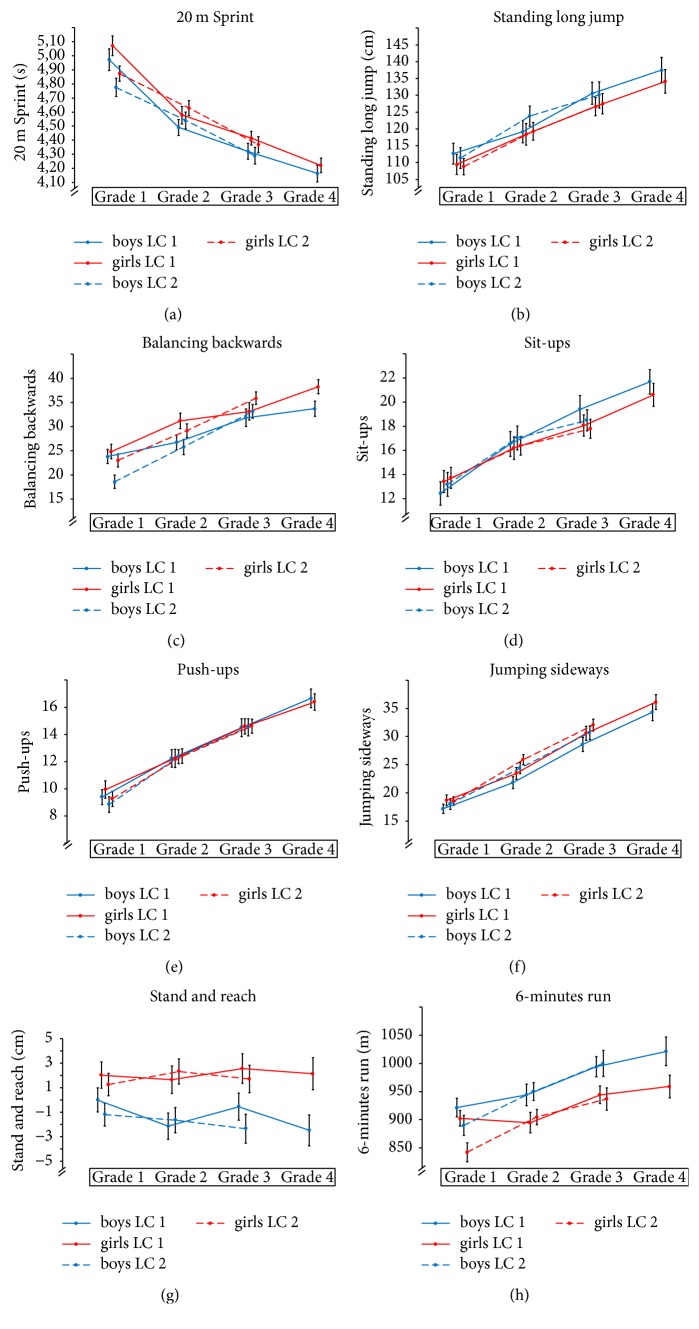
Means and confidence intervals of physical fitness for longitudinal cohort 1 (LC 1) and longitudinal cohort 2 (LC 2).

PF increased significantly over time in all test tasks for both longitudinal cohorts except for stand and reach. Means and confidence intervals of physical fitness for LC 1 and LC 2 are shown in Figures 1(a)–1(h).

In LC 1 the stand and reach performance decreased significantly and significant time*∗*sex interaction shows that the decrease is mainly due to boys (time: *F* = 8.74; *p* < .01; eta^2^ = .034; sex: *F* = 22.82; *p* < .01; time*∗*sex: *F* = 7.95; *p* = <.01; eta^2^ = .017).

Girls achieved better results than boys in balancing backwards (LC 1: *F* = 184.28; *p* =< .01; eta^2^ = .033/LC 2: *F* = 319.87; *p* < .01; eta^2^ = .937), stand and reach (LC 1: *F* = 22.82; *p* =< .01; eta^2^ = .084/LC 2: *F* = 29.07; *p* < .01; eta^2^ = .086), and jumping sideways (LC 1: *F* = 7.19; *p* =< .01; eta^2^ = .028/LC 2: *F* = 3.79; *p* = .05; eta^2^ = .012). Boys achieved better results in the 20 m sprint (LC 1: *F* = 5.69; *p* = .02; eta^2^ = .022/LC 2: *F* = 6.38; *p* = .02; eta^2^ = .020) and in the 6-minute run (LC 1: *F* = 17.90; *p* =< .01; eta^2^ = .029/LC 2: *F* = 115.31; *p* =< .01; eta^2^ = .078).

Additional significant interactions between time and sex were found for LC 1 in sit-ups (*F* = 9.52; *p* < .01; eta^2^ = .037) and 6-minute run (*F* = 7.25; *p* < .01; eta^2^ = .029). In both test tasks, boys showed higher gains than girls. For LC 2, girls showed higher gains than boys in stand and reach (*F* = 5.23; *p* = .02; eta^2^ = .029).

*F*-values (*F*), significance (*p*), and partial eta squared (eta^2^) from rmANOVAS for both longitudinal cohorts (LC 1+ LC 2) are shown in Table 4.

### 3.2. Stability of Motor Performance

A measurement tracked if there was a positive relationship in subjects between measurement points [28]. The magnitude of the correlation coefficient can be estimated according to *r* < 0.30, low stability; *r* = 0.30 to 0.60, moderate; *r* > 0.60, moderately high [14].

Table 5 shows the correlations of the two longitudinal cohorts separated by gender.

All correlations are significant at the level of *p* < .01. On average, boys showed slightly higher correlation coefficients than girls in both cohorts. Moreover, the coefficients for one period are, on average, of moderate stability. The highest correlation coefficients were found from grade 1 to grade 2, with each additional year decreasing.

The results from LC 2 largely confirm those from LC 1. Boys also showed higher coefficients than girls; however the reduction of *r* from grades 1-2 to grades 1–3 was less pronounced.

For both longitudinal cohorts, stand and reach and standing long jump showed the highest stability. The coefficients were constantly above *r* = .50. On the other hand, the correlations for push-ups were poor. Four correlations were below *r* = 0.30 and represented “low stability” according to Malina [14]. Correlations of BMI were considerably higher than correlations of motor tests. The values were higher than *r* = 0.7.

## 4. Discussion

The first goal of the TrieKis study was to present the motor development process of primary schoolchildren for a medium-sized city (Trier; population approximately 115,000) in Germany. The study showed that PF increased significantly over time in all test tasks for both longitudinal cohorts, except for stand and reach.

In the second part of the study, stability of children's physical fitness during primary school period was assessed. PF usually generates higher correlations in comparison to PA [23]. In the course of the study the correlation coefficients decreased slightly, indicating an expected loss of stability when the observed time frame gets larger. Concerning gender differences, boys showed higher correlation coefficients than girls in nearly all test tasks.

### 4.1. Development of PF

PF increased significantly over time in all test tasks and BMI for both longitudinal cohorts, except in stand and reach. The girls in LC 1 and LC 2 achieved better results than boys in balancing backwards, jumping sideways, and stand and reach. The boys of both longitudinal cohorts had better results in the 20 m sprint and in the 6-minute run. These results confirm previous research and show that girls have an advantage in coordination-based tasks and flexibility [8, 9, 29] and boys perform better in conditioning-based test tasks [10, 15, 29]. However, in our study not every conditioning-based test task revealed significantly better performance for boys. For strength-based tasks, sit-ups and push-ups, results were inconsistent. The observed range of age in this study did not include puberty, where boys especially show increased effects from strength-based training [11, 12].

As a result of physical development there was also a recognizable increase of BMI over time in both cohorts (LC 1; *F* = 183.79; *p* < .01; LC 2: *F* = 83.0; *p* < .01).

The results for both longitudinal cohorts are comparable and the second cohort confirms the findings from cohort 1.

Besides the expected overall increase in motor performance during primary school, differentiated gender effects were observed in this study. Although the observed age span did not include puberty, differences in the development of PF between genders were significant (20 m sprint; 6-minute run; stand and reach; jumping sideways; balancing backwards). Assuming that hormonal differences were not yet very pronounced in the observed age span, an explanation could be the fact that boys prefer different sports than girls, mainly due to social pressure. Additionally, even in comparable sports, training content differs between boys and girls [30].

### 4.2. Stability

Regarding the stability of children's PF during primary school, we found decreasing Pearson's correlation coefficients when the observed time span increased. The fact that boys showed higher correlation coefficients than girls has also been found in other studies [10].Overall, the lowest correlations were found for push-ups. This finding may be due to the fact that during the push-up task instructors had to judge correct execution, and since instructors changed every year, this may have led to measurement errors.

Correlations of stand and reach were high and constantly above *r* = 0.50. Correlations of BMI were considerably higher than the correlations of motor tests. This confirmed other study results [16]. The values were always above *r* = 0.7 and “moderately high” [14]. BMI and flexibility are probably quite stable in childhood and adolescence [15]. Falk et al. [10], McMillan and Erdmann [25], and Vandorpe et al. [26] tracked quite similar test groups and also used field-tests in their studies.

Falk et al. [10] examined 319 children (297 boys and 116 girls) from the second to the sixth grade. He found gender-specific differences as in this study. The stability of BMI (boys: *r* = .52; girls: *r* = .47) was clearly lower than in this study (LC1; boys: *r* = .804; girls: *r* = .743). Regarding standing long jump, the correlations (boys: *r* = .43; girls: *r* = .40) in this study were higher for boys (LC 1: boys: *r* = .597). The correlations among the girls were roughly same (LC 1: girls: *r* = .392).

McMillan and Erdmann [25] applied sit-ups and a sit and reach test to check strength and flexibility. Over three years of tracking (Kindergarten-Grade 2; 6.1 ± 0.3 years), they found correlations in sit-ups of *r* = .44 (m.) and *r* = .39 (f.) and in sit and reach *r* = .48 (m.) and .52 (f.). This study found similar and even slightly higher correlations: LC 1: sit-ups: *r* = .548 (m.), *r* = .377 (f.); LC 1: stand and reach: *r* = .531 (m.), *r* = .640 (f.). Flexibility tests seem to be more stable than strength-based tasks. This is also confirmed when the results of LC 2 are compared with McMillan and Erdmann [25].

Concerning the correlations of the coordination tests for a 2-year interval (8–10 years) at primary school age, Vandorpe et al. [26] found better stabilities (*r* > 0.8) than in the TrieKis study (*r* = .428–.512).

Summarizing the results from our study, we have to state that PF in primary school is only of moderate stability. Besides form on the day, rather unstable PA behavior of primary school children could explain this finding. Other studies have shown that PF usually generates higher correlations in comparison to PA [22]. During primary school, many children change their PA from gymnastics or unorganized PA to organized team sports with different training forms and demands [9].

### 4.3. Strength and Limitations of the Study

The DMT 6–18 is a quality-proven test battery that allowed standardized assessment of the PF of the Trier primary school children at 23 different schools. The sample was representative for primary schools of Trier. It was possible to examine motor development and track health-related fitness test data from a large number of boys and girls in two longitudinal cohorts for a 1-, 2-, and 3-year period.

A limitation of the study is that 20 m sprint was measured manually and not by a light-barrier system; therefore measurement inaccuracies may have occurred. Test instructors also changed several times during the course of the study, and hence differences in test execution might have occurred.

A further limitation is that only 40% of the base line took part at all three following measurements, and therefore a bias in measured PF development may have occurred. Furthermore, the second longitudinal cohort only participated from grades 1 to 3 and not from grades 1 to 4.

How the variations in maturation and physical activity levels of participants influenced test results and tracking is unknown. For further investigation, level of activity should be collected annually. In this study, data existed only for the first study year.

Moreover gender differences in performance and development were discussed. These differences change substantial during puberty and we could not rule out the possibility that at least some of the measured children had already reached puberty. In future studies, biological age needs to be assessed in order to interpret gender differences more precisely.

Finally we have to state that the results are not unrestricted generalizable beyond Germany. The age groups in grades of primary schools differ in different countries. Moreover physical education and PA of pupils differ between countries; however the PA of German children and adolescents lies within the average of European countries [31].

### 4.4. Conclusion

Physical fitness increased over the course of the study. Gender-specific differences were obvious, but this could not always be observed (e.g., standing long jump). Consistent with literature, girls are undoubtedly superior in flexibility and boys are better in cardiorespiratory fitness.

The results also demonstrate significant tracking for young school children. Correlations decline with reduced time frames. It is possible that tracking was lower in girls than in boys because of their earlier maturation. In total, moderate stabilities were found. Six out of 90 correlations were only “low” and 17 out of 90 correlations were “moderately high” [14]. Stabilities of BMI were continuously higher than PF.

Studies are difficult to compare due to the variety of fitness tests used, different ages of the subjects at baseline, span of the longitudinal follow-up, and different sample sizes [10, 15]. Variability in the timing and speed of the adolescent growth spurt and sexual maturation will also influence tracking.

## Figures and Tables

**Table 1:** Descriptive statistics ofparticipants of the longitudinal study in Trier, Germany.

Grade	Sex	2008/2009		2009/2010		2010/2011		2011/2012	
		*N*	Age	*N*	Age	*N*	Age	*N*	Age
1	Boys	310	6.86 ± 0.44	358	6.80 ± 0.42				
1	Girls	313	6.80 ± 0.45	381	6.76 ± 0.43				

2	Boys			321	7.84 ± 0.66	337	7.93 ± 0.46		
2	Girls			344	7.81 ± 0.47	360	7.91 ± 0.46		

3	Boys					309	8.95 ± 0.52	234	9.20 ± 0.46
3	Girls					312	8.98 ± 0.50	239	9.09 ± 0.75

4	Boys							204	10.16 ± 0.51
4	Girls							222	10.17 ± 0.52

*Total*		623	6.83 ± 0.45	1404	7.27 ± 0.72	1318	8.41 ± 0.71	899	9.63 ± 0.77

**Table 2:** Mean and standard deviation for longitudinal cohort 1 (LC1) in test tasks and BMI, *N* = 253.

Task	Sex	Grade 1	Grade 2	Grade 3	Grade 4
		(6.8 ± 0.4 years)	(7.8 ± 0.4)	(8.9 ± 0.4)	(10.1 ± 0.4)
		Mean ± s	Mean ± s	Mean ± s	Mean ± s
20 m sprint (sec)	*m*	4.97 ± 0.45	4.50 ± 0.34	4.32 ± 0.34	4.16 ± 0.35
	*f*	5.07 ± 0.38	4.58 ± 0.34	4.41 ± 0.26	4.22 ± 0.28
	∑	5.02 ± 0.42	4.53 ± 0.34	4.36 ± 0.31	4.19 ± 0.32

Standing long jump (cm)	*m*	112.5 ± 18.0	119.0 ± 19.2	130.4 ± 19.0	137.3 ± 21.8
	*f*	109.3 ± 15.8	118.3 ± 16.8	126.5 ± 14.1	134.0 ± 18.2
	∑	111.1 ± 17.1	118.7 ± 18.1	128.7 ± 17.0	135.8 ± 20.3

Balancing backwards	*m*	23.8 ± 8.7	26.7 ± 9.3	31.5 ± 10.6	36.3 ± 9.3
	*f*	24.8 ± 8.1	31.2 ± 8.8	34.5 ± 9.8	38.4 ± 8.0
	∑	24.3 ± 8.4	28.8 ± 9.3	32.8 ± 10.3	37.2 ± 8.8

Sit-ups	*m*	12.5 ± 5.6	16.5 ± 6.1	19.4 ± 6.6	21.7 ± 5.9
	*f*	13.4 ± 4.9	16.2 ± 5.1	18.1 ± 4.8	20.6 ± 5.1
	∑	12.9 ± 5.3	16.4 ± 5.6	18.8 ± 5.8	21.2 ± 5.6

Push-ups	*m*	9.4 ± 3.3	12.2 ± 3.8	14.5 ± 3.9	16.6 ± 4.0
	*f*	9.9 ± 3.5	12.3 ± 3.6	14.6 ± 3.0	16.4 ± 3.3
	∑	9.6 ± 3.4	12.2 ± 3.7	14.5 ± 3.5	16.5 ± 3.7

Jumping sideways	*m*	17.2 ± 4.8	21.8 ± 6.1	28.6 ± 7.2	34.3 ± 8.6
	*f*	18.7 ± 5.2	23.5 ± 5.7	30.6 ± 6.9	36.1 ± 7.2
	∑	17.9 ± 5.0	22.6 ± 6.0	29.5 ± 7.1	35.1 ± 8.0

Stand and reach (cm)	*m*	0.0 ± 5.8	−2.1 ± 6.3	−0.5 ± 6.4	−2.5 ± 7.4
	*f*	2.0 ± 5.9	1.6 ± 6.1	2.6 ± 6.6	2.1 ± 7.1
	∑	0.9 ± 5.9	−0.4 ± 6.5	0.9 ± 6.7	0.9 ± 6.7

6-minute run (m)	*m*	921.4 ± 97.3	944.4 ± 112.7	994.1 ± 105.5	1021.6 ± 148.9
	*f*	902.4 ± 74.4	895.0 ± 97.0	944.3 ± 82.5	959.0 ± 106.6
	∑	912.8 ± 88.1	922.1 ± 108.6	971.6 ± 98.8	993.3 ± 134.9

BMI	*m*	15.9 ± 2.2	16.6 ± 2.3	16.9 ± 3.0	17.9 ± 3.0
	*f*	15.5 ± 1.7	16.1 ± 1.9	16.3 ± 2.1	17.5 ± 2.5
	∑	15.7 ± 2.0	16.4 ± 2.2	16.6 ± 2.6	17.7 ± 2.8

**Table 3:** Mean and standard deviation for longitudinal cohort 2 (LC 2) in test tasks and BMI, *N* = 315.

Task	Sex	Grade 1	Grade 2	Grade 3
		(6.7 ± 0.4 years)	(7.8 ± 0.4)	(9.1 ± 0.4)
		Mean ± s	Mean ± s	Mean ± s
20 m sprint (sec)	*m*	4.78 ± 0.40	4.54 ± 0.37	4.29 ± 0.37
	*f*	4.87 ± 0.35	4.63 ± 0.35	4.37 ± 0.36
	∑	4.83 ± 0.37	4.59 ± 0.36	4.33 ± 0.37

Standing long jump (cm)	*m*	111.2 ± 18.5	123.7 ± 17.9	130.0 ± 22.8
	*f*	108.7 ± 15.6	119.2 ± 17.0	127.4 ± 19.6
	∑	109.9 ± 17.0	121.3 ± 17.5	128.6 ± 21.2

Balancing backwards	*m*	18.6 ± 8.7	25.7 ± 9.7	33.1 ± 8.8
	*f*	23.0 ± 9.0	29.2 ± 9.4	35.9 ± 8.6
	∑	20.9 ± 9.1	27.5 ± 9.7	34.5 ± 8.8

Sit-ups	*m*	13.2 ± 6.1	17.1 ± 6.0	18.5 ± 5.3
	*f*	13.7 ± 5.7	16.4 ± 5.0	17.8 ± 5.2
	∑	13.5 ± 5.9	16.7 ± 5.5	18.1 ± 5.3

Push-ups	*m*	8.8 ± 3.6	12.4 ± 3.2	14.5 ± 3.8
	*f*	9.3 ± 3.6	12.4 ± 3.5	14.5 ± 3.8
	∑	9.1 ± 3.6	12.4 ± 3.4	14.6 ± 3.6

Jumping sideways	*m*	18.0 ± 5.9	24.4 ± 6.2	30.7 ± 7.4
	*f*	18.5 ± 4.9	25.9 ± 6.0	32.0 ± 6.7
	∑	18.3 ± 5.4	25.2 ± 6.1	31.4 ± 7.1

Stand and reach (cm)	*m*	−1.2 ± 5.7	−1.6 ± 6.3	−2.3 ± 7.2
	*f*	1.3 ± 5.9	2.3 ± 6.8	1.7 ± 7.3
	∑	0.1 ± 5.9	0.5 ± 6.8	−0.2 ± 7.5

6-minute run (m)	*m*	890.0 ± 108.0	950.1 ± 97.3	1000.0 ± 141.6
	*f*	842.3 ± 109.5	905.1 ± 87.5	937.0 ± 126.2
	∑	865.0 ± 111.2	926.5 ± 94.8	967.0 ± 137.2

BMI	*m*	16.3 ± 2.6	16.2 ± 2.5	17.1 ± 2.7
	*f*	15.9 ± 1.8	16.0 ± 2.2	17.0 ± 2.6
	∑	16.1 ± 2.2	16.1 ± 2.3	17.0 ± 2.7

**Table 4:** rmANOVA results for longitudinal cohort 1 (LC 1) and longitudinal cohort 2 (LC 2).

Task	LC (start year)	Effects	*F* =	*p* =	Eta^2^ =
20 m sprint (sec)	LC 1 (2008)	Time	552.18	<.01	.688
		Sex	5.69	.02	.022
		Time*∗*sex	0.66	.42	.003
	LC 2 (2009)	Time	336.93	<.01	.518
		Sex	6.38	.02	.020
		Time*∗*sex	0.25	.62	.001

Standing long jump (cm)	LC 1 (2008)	Time	207.79	<.01	.467
		Sex	2.05	.15	.009
		Time*∗*sex	0.19	.66	.001
	LC 2 (2009)	Time	173.29	<.01	.369
		Sex	3.14	.08	.011
		Time*∗*sex	0.002	.968	.000

Balancing backwards	LC 1 (2008)	Time	184.28	<.01	.423
		Sex	8.48	<.01	.033
		Time*∗*sex	0.20	.66	.001
	LC 2 (2009)	Time	319.87	<.01	.505
		Sex	19.11	<.01	.937
		Time*∗*sex	2.64	.11	.008

Sit-ups	LC 1 (2008)	Time	218.54	<.01	.469
		Sex	0.598	.44	.002
		Time*∗*sex	9.52	<.01	.037
	LC 2 (2009)	Time	109.63	<.01	.261
		Sex	0.29	.59	.001
		Time*∗*sex	3.48	.06	.011

Push-ups	LC 1 (2008)	Time	270.53	<.01	.523
		Sex	0.09	.77	.000
		Time*∗*sex	1.84	.18	.007
	LC 2 (2009)	Time	276.31	<.01	.472
		Sex	0.35	.55	.001
		Time*∗*sex	0.54	.46	.002

Jumping sideways	LC 1 (2008)	Time	630.16	<.01	.717
		Sex	7.19	<.01	.028
		Time*∗*sex	0.12	.95	.000
	LC 2 (2009)	Time	1291.64	<.01	.807
		Sex	3.79	.05	.012
		Time*∗*sex	1.34	.25	.004

Stand and reach (cm)	LC 1 (2008)	Time	8.74	<.01	.034
		Sex	22.82	<.01	.084
		Time*∗*sex	7.95	<.01	.031
	LC 2 (2009)	Time	2.08	.13	.007
		Sex	29.07	<.01	.086
		Time*∗*sex	5.23	.02	.017

6-minute run (m)	LC 1 (2008)	Time	61.52	<.01	.200
		Sex	17.90	<.01	.068
		Time*∗*sex	7.25	<.01	.029
	LC 2 (2009)	Time	115.31	<.01	.273
		Sex	25.90	<.01	.078
		Time*∗*sex	1.04	.36	.003

BMI	LC 1 (2008)	Time	183.79	<.01	.423
		Sex	13707.97	.09	.011
		Time*∗*sex	0.04	.83	.000
	LC 2 (2009)	Time	83.0	<.01	.211
		Sex	1.12	.29	.004
		Time*∗*sex	2.99	.09	.010

**Table 5:** Pearson correlation coefficients (*r*) of test tasks and BMI.

Longitudinal cohort 1	Boys (*N* = 137)		
Test task	Grades 1-2	Grades 1–3	Grades 1–4

20 m sprint	.552^*∗*^	.633^*∗*^	.569^*∗*^
Standing long jump	.635^*∗*^	.592^*∗*^	.597^*∗*^
Balancing backwards	.589^*∗*^	.499^*∗*^	.444^*∗*^
Push-ups	.376^*∗*^	.328^*∗*^	.311^*∗*^
Sit-ups	.679^*∗*^	.534^*∗*^	.548^*∗*^
Jumping sideways	.489^*∗*^	.469^*∗*^	.317^*∗*^
Stand and reach	.593^*∗*^	.590^*∗*^	.531^*∗*^
6-minute run	.476^*∗*^	.470^*∗*^	.499^*∗*^
BMI	.845^*∗*^	.774^*∗*^	.804^*∗*^

	Girls (*N* = 115)		

Test task	Grades 1-2	Grades 1–3	Grades 1–4

20 m sprint	.494^*∗*^	.456^*∗*^	.340^*∗*^
Standing long jump	.624^*∗*^	.500^*∗*^	.392^*∗*^
Balancing backwards	.453^*∗*^	.438^*∗*^	.376^*∗*^
Push-ups	.414^*∗*^	.276^*∗*^	.279^*∗*^
Sit-ups	.585^*∗*^	.438^*∗*^	.377^*∗*^
Jumping sideways	.501^*∗*^	.428^*∗*^	.268^*∗*^
Stand and reach	.616^*∗*^	.655^*∗*^	.640^*∗*^
6-minute run	.429^*∗*^	.517^*∗*^	.339^*∗*^
BMI	.880^*∗*^	.726^*∗*^	.743^*∗*^

Longitudinal cohort 2	Boys (*N* = 149)		

Test task	Grades 1-2	Grades 1–3	

20-meter sprint	.604^*∗*^	.632^*∗*^	
Standing long jump	.535^*∗*^	.568^*∗*^	
Balancing backwards	.323^*∗*^	.467^*∗*^	
Push-ups	.297^*∗*^	.331^*∗*^	
Sit-ups	.523^*∗*^	.480^*∗*^	
Jumping sideways	.583^*∗*^	.512^*∗*^	
Stand and reach	.576^*∗*^	.512^*∗*^	
6-minute run	.625^*∗*^	.565^*∗*^	
BMI	.727^*∗*^	.707^*∗*^	

	Girls (*N* = 166)		

Test task	Grades 1-2	Grades 1–3	

20 m sprint	.522^*∗*^	.550^*∗*^	
Standing long jump	.599^*∗*^	.503^*∗*^	
Balancing backwards	.548^*∗*^	.433^*∗*^	
Push-ups	.235^*∗*^	.356^*∗*^	
Sit-ups	.414^*∗*^	.411^*∗*^	
Jumping sideways	.456^*∗*^	.481^*∗*^	
Stand and reach	.624^*∗*^	.615^*∗*^	
6-minute run	.342^*∗*^	.261^*∗*^	
BMI	.878^*∗*^	.897^*∗*^	

^*∗*^ *p* = <.01.

## Abbreviations

BMI: Body mass index
DMT 6–18: German Motor Test 6–18
PA: Physical activity
PF: Physical fitness.
